# Department Wide Validation in Digital Pathology—Experience from an Academic Teaching Hospital Using the UK Royal College of Pathologists’ Guidance

**DOI:** 10.3390/diagnostics13132144

**Published:** 2023-06-22

**Authors:** Mai Kelleher, Richard Colling, Lisa Browning, Derek Roskell, Sharon Roberts-Gant, Ketan A. Shah, Helen Hemsworth, Kieron White, Gabrielle Rees, Monica Dolton, Maria Fernanda Soares, Clare Verrill

**Affiliations:** 1 Department of Cellular Pathology, John Radcliffe Hospital, Oxford University Hospitals NHS Foundation Trust, Oxford OX3 9DU, UK; 2 Nuffield Department of Surgical Sciences, Oxford University, Oxford OX3 9DU, UK; 3 NIHR Oxford Biomedical Research Centre, Oxford University Hospitals NHS Foundation Trust, Oxford OX3 9DU, UK

**Keywords:** digital pathology, validation, Royal College of Pathologists, department-wide, discordances, digital whole slide images, diagnostic confidence, artificial intelligence, stage 2 validation

## Abstract

Aim: we describe our experience of validating departmental pathologists for digital pathology reporting, based on the UK Royal College of Pathologists (RCPath) “Best Practice Recommendations for Implementing Digital Pathology (DP),” at a large academic teaching hospital that scans 100% of its surgical workload. We focus on Stage 2 of validation (prospective experience) prior to full validation sign-off. Methods and results: twenty histopathologists completed Stage 1 of the validation process and subsequently completed Stage 2 validation, prospectively reporting a total of 3777 cases covering eight specialities. All cases were initially viewed on digital whole slide images (WSI) with relevant parameters checked on glass slides, and discordances were reconciled before the case was signed out. Pathologists kept an electronic log of the cases, the preferred reporting modality used, and their experiences. At the end of each validation, a summary was compiled and reviewed with a mentor. This was submitted to the DP Steering Group who assessed the scope of cases and experience before sign-off for full validation. A total of 1.3% (49/3777) of the cases had a discordance between WSI and glass slides. A total of 61% (30/49) of the discordances were categorised as a minor error in a supplementary parameter without clinical impact. The most common reasons for diagnostic discordances across specialities included identification and grading of dysplasia, assessment of tumour invasion, identification of small prognostic or diagnostic objects, interpretation of immunohistochemistry/special stains, and mitotic count assessment. Pathologists showed similar mean diagnostic confidences (on Likert scale from 0 to 7) with a mean of 6.8 on digital and 6.9 on glass slide reporting. Conclusion: we describe one of the first real-world experiences of a department-wide effort to implement, validate, and roll out digital pathology reporting by applying the RCPath Recommendations for Implementing DP. We have shown a very low rate of discordance between WSI and glass slides.

## 1. Introduction

Digital pathology (DP), reporting with whole slide images (WSI) created from high-resolution scans of glass slides, has the potential to improve the quality, safety, and efficiency of diagnostic pathology [1,2,3]. Few UK departments have fully transitioned to primary DP, but as part of the National Health Service (NHS) long term plan for digitally enabled care, more are embarking on this journey [4]. This technological modernisation could future-proof histopathological services. At a time of huge workforce shortages and growing pressure to improve diagnostic accuracy and turnaround times [5,6], it will also likely pave the way for the use of artificial intelligence (AI) algorithms, such as automated image analysis to support diagnostic assessments and provide novel insights into disease processes [7].

Any novel technology will be met with a combination of enthusiasm and doubt, and pathologists vary in their computer literacy and aversion to risk. Prior to transitioning to DP in our department, the viewpoints of our pathologists, biomedical scientists, and administrative team were surveyed [8]. Anticipated benefits included improvements in collaboration, teaching, cost saving, research, speciality multidisciplinary teams, and patient-centred care. Key barriers to digitisation were thought to be standardisation, validation, national implementation, storage and backup, training, logistical implementation, cost-effectiveness, privacy, and legality [8].

The Department of Cellular Pathology at the Oxford University Hospitals NHS Foundation Trust (OUHFT) is one of the first cellular pathology departments in the UK to undergo DP transformation. We are part of the PathLake consortium, one of five UK government funded Centres of Excellence in DP and medical imaging, which aims to reach full digitisation of participating cellular pathology laboratories [9]. The COVID-19 pandemic accelerated the roll out and adoption of DP as it was particularly valuable in allowing pathologists to work remotely [10]. OUHFT achieved 100% digitisation of surgical slides for diagnostic reporting in 2020.

A period of validation is required before pathologists may report digitally, and guidelines released by the College of American Pathologists (CAP) and the UK Royal College of Pathology (RCPath) provide a useful framework [11,12]. The CAP validation guidelines advise that at least 60 cases are viewed on digital and glass, with a suggested washout period of at least 2 weeks between viewing digital and glass slides, and a rate of diagnostic concordance greater than 95% [11].

In contrast to CAP guidelines, RCPath “Best Practice Recommendations for Implementing Digital Pathology” highlights the importance of validation in a “real world” setting [12]. It focuses on professional development of the individual and the acquisition of competence [13]. Four suggested phases are described: (1) basic skills training—formalised training with the DP platform and supervised practice, (2) Stage 1 validation—retrospective review of a training set of cases, representative of the pathologist’s usual scope of work, (3) Stage 2 validation—prospective reporting of digital cases with reference to glass slides for at least the highlights/relevant parameters to confirm concordance on all diagnostic areas and build confidence, and (4) validation document with recommendations and on-going monitoring. 

In this study, we share the method used for Stage 2 validation, our experiences, and outcomes. The learning curves and challenges are highlighted. Our experience of basic skills training and Stage 1 validation is beyond the scope of this paper and has been published previously [13,14,15]. To the best of our knowledge, this is one of the few reports of department-wide efforts in validation using the RCPath guidelines and includes collated real-time reflections on DP upskilling, caveats, and interface user experience across various specialities.

## 2. Materials and Methods

### 2.1. Digitisation of Surgical Pathology in the Laboratory

This study was performed in the Department of Cellular Pathology of an academic teaching hospital in Southeast England. Hardware comprised five Philips slide scanners (four ultrafast and one ultra-versatile) linked to Philips Image Management System. Standard slides are scanned with a 40× objective, and large slides with 20×. More than 1500 WSI and 1.6 terabytes of data are produced each day, and the yearly output is approximately 335,000 surgical histology and immunohistochemistry slides, 4000 extra-large slides, and approximately 33,000 referral slides. Full digital deployment does not currently include post-mortem slides or cytology preparations, and only some referrals are digitised by speciality. The laboratory has accreditation (extension to scope) under the United Kingdom Accreditation Service (ISO:15189) for DP. Further details of the digisation of our central laboratory and of its network are outlined elsewhere [16].

### 2.2. Validation for Full DP Reporting

Twenty histopathologists completed Stage 2 validation for DP, between the 17th of January 2019 and the 29th of July 2022. This covered eight specialities (Table S1): Urology (Urology Germ cell—UGC, Bladder Kidney Penile—BKP, and Prostate), Breast, Renal, Gastrointestinal (GI, and Liver), Head and Neck (H&N), Skin, Gynaecology (Gynae), and Respiratory. Haematopathology, Neuropathology, and Bone/Soft Tissue pathologists were excluded due to local logistics. The twenty participating histopathologists included newly qualified junior consultants to specialists with over 10 years of experience in one or multiple areas. Trainee pathologists were not included in this analysis.

The pathologists undertook the validation process for full DP reporting as per the RCPath guidelines with a few alterations to meet recommendations of the local governance group [12]. As per the RCPath guidance and local DP validation SOP, mandatory glass check of relevant parameters in every case was undertaken after reviewing the digital images. An additional local requirement for Stage 2 validation was for pathologists to review all digital and glass slides of at least 20 multi-block cases, rather than just relevant parameters. This aimed at understanding how pathologists navigate through a large number of blocks, and if digital slides predispose pathologists to quickly flick through WSIs at low power.

Records on all cases were logged in a bespoke electronic form inbuilt within the FileMaker Pro database. The electronic form included information on the date the case was received and the date it was signed out; whether digital and/or glass was preferred for primary reporting; and the diagnostic confidence on digital versus glass on a 0–7 Likert Scale. In order to predict appropriate judgement on the need to view glass after digital once fully validated, Pathologists were also asked to speculate on whether they would defer to glass slides before signing the case out, or if they would be confident to authorise the case on digital images alone. Any discordance in the cases between digital and glass were also recorded. The discordances were classified into B1—serious error with fundamental aspects of the case, B2—significant error in supplementary parameter, B3—minor error in supplementary parameter, or N/A—no clinical impact [17]. Deferrals to glass for technical reasons were recorded as a category A error and not included in discordances as these were technical issues. These observations allowed the generation of a technical pitfall checklist, including out of focus slides, failed calibration, tissue out of the field of scanning, missed tissue (often pale fatty tissue), suboptimal staining, and missed WSIs within a multi-block case.

Histopathologists identified as “early adopters” or those with prior DP experience were identified as DP mentors and supported other pathologists through validation. Learning points acquired during validation, such as the “potential pitfalls” of digital reporting were shared regularly within the department. A DP Steering Group, established during the implementation phase, remained active through the whole validation and roll-out process and assessed its progress, dealt with difficulties and achievements, and made sure the RCPath guidelines were abided by. The local departmental Governance Group had oversight of DP full validation sign-off. Full validation sign-off required the summary of all submitted Stage 2 forms to be verified by the speciality lead, followed by submission of the summary to the DP Steering Group. If range and suitability requirements were not met, either the DP lead or a DP mentor would discuss the caveats, ensure understanding of pitfalls, and advise on potential mitigating measures. The verified summaries submitted by the 20 participating histopathologists were the source material for this paper.

## 3. Results

**Figure 1:**
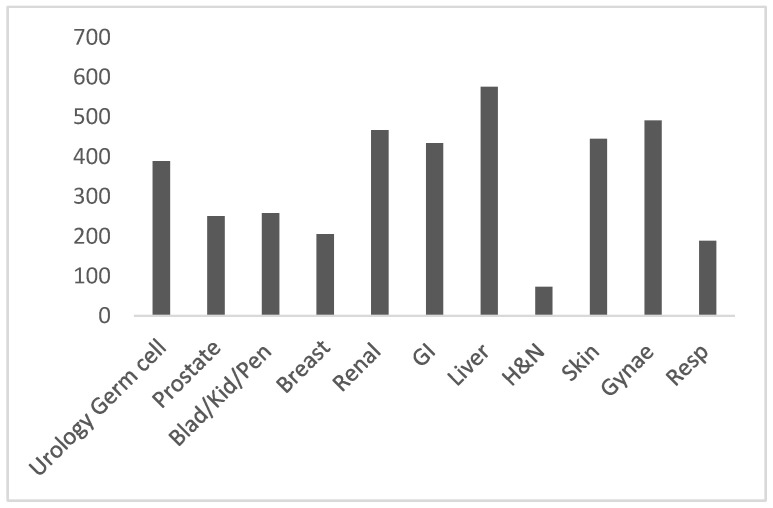
Average number of days taken for validation per pathologist by specialty.

Pathologists took a mean of 362 days (11.9 months) and a median of 381 days (12.5 months, range 10–793 days) to complete Stage 2 validation (Table 1, Figure 1).

A total of 3777 cases were viewed, with 100% cases being viewed digitally and then with a glass check, with a mean average of 135 cases viewed per pathologist. The specialities varied in the number of cases reported during validation. The lowest and highest number of cases needed for Stage 2 validation was 50 and 242 cases for histopathologists reporting UGC and skin, respectively (Table 2).

**Figure 2:**
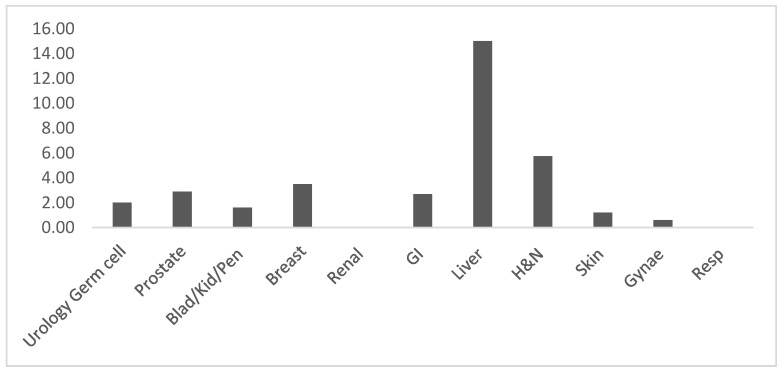
Deferred to glass due to a technical issue (% of cases).

During validation, an average technical failure rate of 2.6% was encountered, with Liver being the speciality that most often encountered technical issues (15/99 cases) (Figure 2). These hardships were mostly due to out of focus areas and difficulties in interpreting the orcein stain on WSIs due to a perceived lower contrast compared to glass slides (Table S2). These difficulties tended to become less reported or were resolved over time. Other technical issues were related to scanning or to interaction with the image viewing interface. In only 0.1% of cases (3/3777) a poor-quality digital image led to deferral to glass (such a technical issue was categorised as A). Of note, the focus in this validation process was on diagnostic discordance, and thus this should not necessarily be considered an absolute rate of technical issues. Most technical issues did not stop the pathologist from reporting the case digitally but sometimes caused delay due to rescanning or interface malfunction. Anecdotally, most technical issues encountered resolved over time with improvements in workflow, bandwidth arrangements, development of more robust pipelines, and with departmental learning.

### 3.1. Cases with Discordances

A total of 1.3% of cases (49/3777) dual reported on glass and digital (range: none—Gynaecology, H&N, and Respiratory to 5.3% of cases—BKP) (Figure S1). This does not include those caused by technical issues (category A) which are counted separately, as outlined above (*n* = 3). Most discordances (30/49 cases) were of no clinical impact. Where potential harm could have ensued (19/49 cases), this was due to a minor error in a supplementary parameter in 16 cases (B3 error, Table 3). In three cases, there was a discordance that implied a significant error in a supplementary parameter (B2 error); in all these three instances, in recognition that these were likely challenging cases, the pathologists stated that once fully validated they would have checked glass slides before authorizing the case. Two of the cases were bladder biopsies, one in which a significant amount of inflammation made it difficult to assess dysplasia on WSIs, and another was a case of a urothelial carcinoma in which grading was not readily clear on digital slides. The final B2 error was a missed micrometastasis in a pericolonic lymph node; this did not change the staging as other nodal metastases had already been identified. 

**Figure 3:**
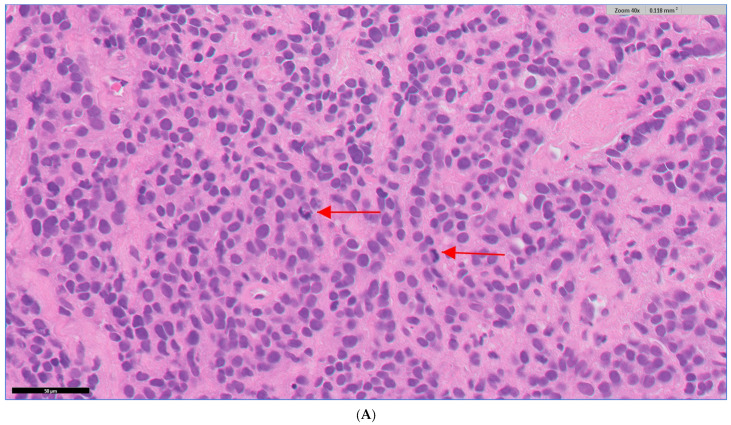

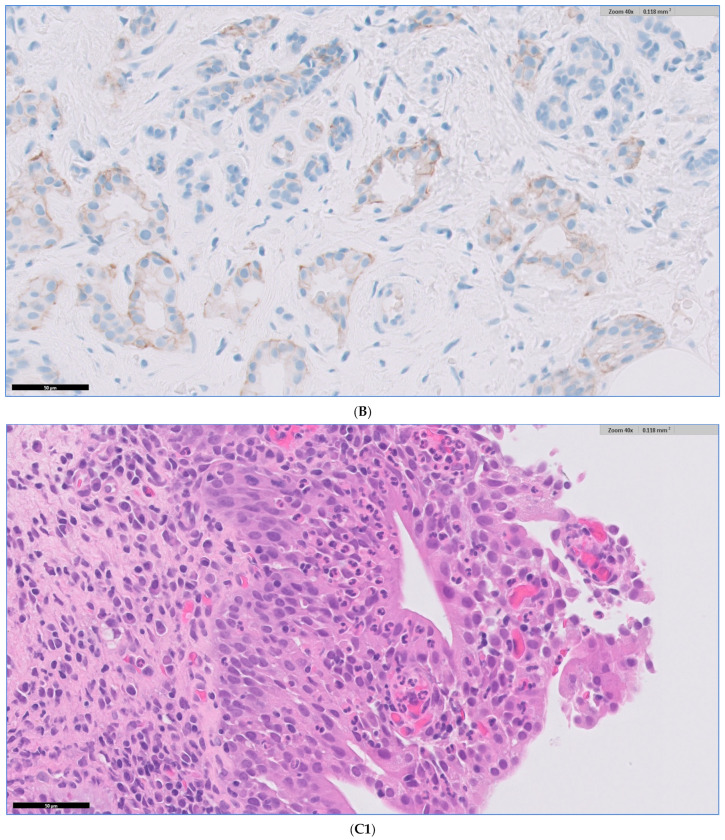

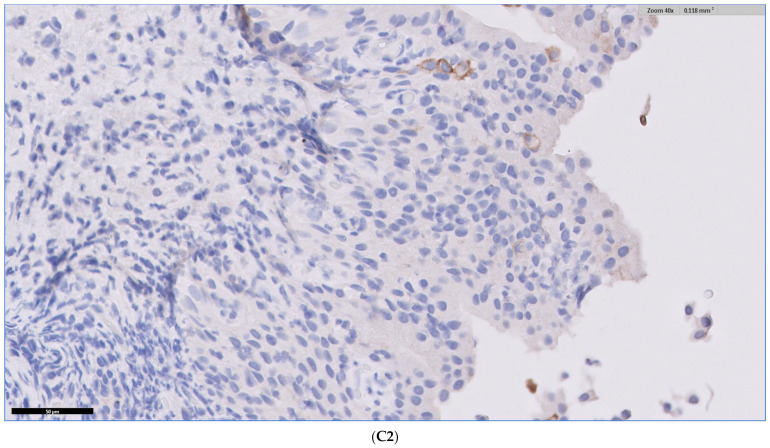
Examples of histopathological features that led to discordances. (**A**) Mitotic figures in a breast carcinoma biopsy (invasive carcinoma with ductal phenotype) were easier to see on glass with digital being poor at high power. The carcinoma was easier to grade (as grade 3) on glass. Arrows highlight examples of mitotic figures. (**B**) A HER-2-stained breast core biopsy containing breast carcinoma. It was assessed as just reaching 2+ on digital (with low confidence) but on glass check was less intense and considered a 1+ category. The pathologist highlighted that they were aware of difficulty in assessing these cases on digital and would seek more practice and experience. (**C1**): A bladder biopsy showing florid reactive changes. It was easier on glass to be more confident that the changes were reactive rather than neoplastic with the pathologist reflecting that the atypia stands out more on digital. (**C2**) CK20 staining was not full thickness, staining umbrella cells only and being supportive of reactive changes.

Causes for discordances varied within specialities (Figure 3 and Table 4) but common themes included identification and grading of dysplasia (12 cases; Prostate, BKP, and Breast), assessment of small areas of tumour invasion (three cases; BKP, GI, and Liver), mitotic count assessment (4 cases; Breast and Skin), and diagnosis of unusual or complex cases (three cases; BKP and Renal). Identification of small prognostic or diagnostic objects was another common cause for discordance (eight cases; Prostate, BKP, Breast, Renal, GI, Liver, and Skin) and specific examples include identification of micro-organisms (Renal, GI, and Liver), weddellite calcification (Breast), metastasis or micrometastasis in lymph nodes (GI and Prostate), and small deposits of amyloid (Renal and Skin) and mucin (Breast and GI). Interpretation of immunohistochemistry and special stains led to discrepancies in four cases (Breast and GI). This included assessment of toluidine blue stain (GI) and immunohistochemistry for Her2 (Breast). To the best of our knowledge, these examples have not been previously reported in the literature. 

### 3.2. Potential Pitfalls of Digital Reporting That Did Not Cause Discordance

Several features on the digital platform were found to be challenging by pathologists, although they did not result in a discordance (Table S3). The most common potential pitfalls included interpretation of special stains in suspected infection (20 comments; Renal, GI, and Liver), differentiation of reactive atypia from dysplasia in an inflammatory background (17 comments; BKP and Breast), identification of mitoses (15 comments; Breast, Skin, GI, Liver, Gynaecology, and Respiratory), and identification of necrosis (six comments; BKP, Renal, Liver, and Gynaecology). A tendency to over-diagnose low-grade dysplasia was noted (nine comments; BKP, Breast, and GI). Less widely reported pitfalls included identification of spermatogonia (10 comments; UGC), identification of spikes and lucencies on silver stain (three comments; Renal), giant cells (one comment; Respiratory), foreign body material (one comment; BKP), and Paneth cell/intestinal metaplasia (two comments; GI). Interface changes were felt to be harder to identify digitally in skin WSI (two comments).

Histopathologists offered varying perspectives on some parameters: whereas lymphovascular invasion in germ cell tumour of the testis was found to be more easily detected on the digital platform by some UGC pathologists (three comments), other UGC pathologists found it more challenging digitally (two comments). Similarly, detection of perineural invasion was considered more difficult in prostate and skin WSI (three comments), and easier in H&N cases (one comment), when compared to their glass counterparts. 

### 3.3. Learning Curves over Time

Some of the challenges encountered during Stage 2 validation were considered part of a learning curve and improved over time (Table 5).

### 3.4. Diagnostic Areas That Potentially May Be Easier on the Digital Platform

Forty-four comments were recorded reporting a preference for the low power and wide field overview available on the digital platform, as it made it easier to pan over the slide (Prostate, UGC, Skin), to assess tumour distribution (Prostate) and vascular invasion (Liver and UGC) (Table S4). A small biopsy assessment was found to be easier and quicker (three comments; BKP and Skin). Certain small diagnostic features were also easier to visualise on digital (18 comments) such as inflammatory cells (GI), ova (BKP), viral inclusions (Renal), oxalate crystals (Renal), *Candida* sp. and pinworms (GI), and megamitochondria (Liver). Interpretation of some stains was easier on DP (15 comments) including interpretation of some grades of Her2 status (Breast), and silver stain for identification of glomerular basement membrane ruptures (Renal). A perceived starker contrast of immunohistochemistry stains on WSI was helpful in the interpretation of mismatch repair (MMR) protein and C4d staining (three comments), particularly when it appeared weak/doubtful on glass.

### 3.5. Pathologists’ Experiences of Digital Reporting

The digital interface played an important role in the pathologists’ experience of digital reporting (Table S5). The features most quoted as helpful on digital slides were the ability to take measurements more quickly and precisely (90 comments) and the low magnification view (38 comments). Pathologists commented that caution is needed, however, with low-power overview as it can lead to false assurance and slides must still be reviewed carefully to avoid missing high-power features (one comment). The importance of carefully scanning the slide on WSI as is done on glass slides was emphasised in dermatopathology for review of melanocytic lesions in particular.

Other significant advantages were pointed out, such as drafting the report on the screen whilst simultaneously looking at the digital slides (13 comments) and being able to simultaneously visualise H&E slides alongside immunohistochemistry for easy comparison (12 comments; Urology, GI, and Gynaecology). Another common theme was how DP facilitated double reporting (21 comments), including working with and teaching trainees (two comments) and how remote working was made possible (two comments). 

The digital interface allowed cases to be shown to clinicians (seven comments), and a renal pathologist stated it to be “an extraordinary asset for multidisciplinary team and individual case discussion and allowed us to keep going in COVID-19 times”.

A total of 38 comments were made regarding the time-consuming effort of screening large areas using a mouse (Table S6). These complaints tended to become less frequent over time. One pathologist reported wrist pain from scanning extra-large blocks; a 3D mouse was supplied, and it helped to relieve these symptoms.

### 3.6. Diagnostic Confidence and Diagnostic Modality Preference

**Figure 4:**
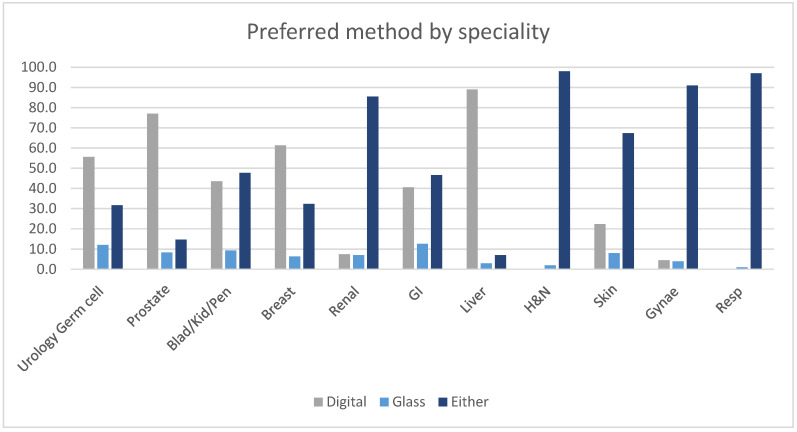
Preferred method of pathologists by speciality.

Across specialities, pathologists showed similar mean diagnostic confidences (on a Likert scale from 0 to 7)—a mean of 6.8 on digital and 6.9 on glass slide reporting (Table S7). A total of 41% of pathologists preferred to report using the digital platform or had no preference between reporting modalities (51%; Figure 4 and Table S8). The minority of cases in which glass reporting was preferred (8%) included those with large tissue areas for scanning, mitotic counting, grading of dysplasia when borderline, and identification of subtle microorganisms. Glass reporting was also preferred when diagnosis required polarisation (Skin and Renal in particular), and for unusual or challenging cases.

## 4. Discussion

To the best of our knowledge, this is the first description of the real-time experience of a pan-department implementation of DP validation using the UK RCPath guidance [12]. Overall, the guidance was found to be appropriate and easily applied.

### 4.1. Engagement and Time to Validation

Not every consultant who was eligible for Stage 2 validation completed the process within the lead time, which is a limitation of this study. This reflects the reality of Histopathology departments, where some pathologists may struggle more than others to complete the validation process due to lack of time, aversion to novelty, logistical constraints, or personal circumstances. Departments should consider how to overcome these challenges, by, for example, including Stage 2 validation as part of the yearly personal development plan, and actively chasing the completion of the process.

There was great variation in the time taken to complete Stage 2 and to obtain a fully validated status for DP reporting (Table S9). The shortest time (10 days) was recorded for a pathologist who submitted a log of 19 cases when applying for validation to report prostate core biopsies. Although this appears to conflict with the 60-case tally suggested by the College of American Pathologists, the 19-case figure is representative of approximately one months’ worth of workload for such specimens and is in keeping with the RCPath suggestions of 1–3 months full-time equivalent practice [11,12]. In contrast, the longest time (793 days) was taken by a Gynaecology pathologist due to a year’s gap. It is worth noting that the bulk of our observations coincided with the COVID-19 pandemic, which led to a discontinuous inflow of some specimens and variable engagement with the validation process.

Although the figures above may suggest leniency, these data reflect a real-world environment. The validation set does not necessarily represent consecutive cases or continuous periods of practice, and some pathologists underwent validation in multiple specialities simultaneously. This further underlines the importance of flexibility to achieve validation.

### 4.2. Number of Cases Needed for Stage 2 Validation

UGC was the speciality with the lowest average of cases needed for sign-off (Table S9). This is in keeping with UGC as a small component of Urology with relatively rare tumour types. This contrasts to Skin, where the average of 242 cases reflects the higher case volume of routine specimens with a wide range of variability [18]. During validation, the Skin team felt that the range of specimens was more important than the numbers to achieve the fully validated status. The need to include a spectrum of skin lesions, for example, melanocytic and non-melanocytic tumours, and inflammatory dermatoses for a thorough DP validation process, has been highlighted by dermatopathologists undergoing validation elsewhere [18,19]. 

### 4.3. Causes of Discordance and Mitigating Strategies

The discordances that arose were relatively few and none were serious. Most consisted of previously described pitfalls [2,20].

Grading of dysplasia is one of the most common causes of clinically significant discordances in DP, across different specialities [2]. Discordance can be caused by missing a focus of dysplasia on the initial low-power review and blurring of nuclear detail when at high power [21]. Assessment of dysplasia on the digital platform has been shown to improve with experience as the features are correlated on digital with glass [22]. This is a key area for the pathologist undergoing validation in DP, and self-validation with glass checks is strongly advised when dysplasia is diagnosed on WSI [2].

Identification of mitoses was a common potential pitfall, raised by Breast, Skin, GI, Liver, Gynaecology, and Respiratory pathologists. Most used the 0.2 mm^2^ grid function available digitally for counting mitoses, which correlates to 0.50/0.51 mm field diameter scores. A glass check is advised for mitotic count, particularly when there is any uncertainty or if the mitotic count is at a borderline value that can lead to clinical impact [21]. This was particularly highlighted as necessary by departmental GI and Respiratory pathologists when facing cases of neuroendocrine and gastrointestinal stromal tumours, as described. Of note, there is a tendency for guidelines to move from assessment of features per power field(s) to millimetre square to accommodate the transition from glass to digital. The assessment of somatic type malignancy in Teratoma, Post Pubertal Type exemplifies this [23].

An important cause of diagnostic discordance is the identification of small diagnostic and prognostic objects which are often eosinophilic and refractile due to the technical nature of the scans. DP also lacks the depth of field (to a degree) available on glass and cannot be polarized [2,20,21]. Future mitigating strategies for these may include scanners capable of Z-plane stacking, focus blending techniques, opting to scan/re-scan sections on higher magnification, or higher definition settings where needed.

Some difficulties in interpreting immunohistochemistry and special stains reported by us are in keeping with prior literature [21]. Our group has, however, highlighted potential challenging areas that appear not to have been acknowledged in the precursory literature. Our consensus is that decisions depending on these should be deferred to glass slides. 

Finally, the three B2 errors recorded over the whole validation process led us to elaborate on mitigating measures. Of note, two cases referred to problematic identification and grading of urothelial carcinoma. This is a known area of potential challenge on DP, and histopathologists are strongly advised to consider deferring this decision to glass slides when challenging [15]. In the case of the missed micrometastasis, the histopathologist involved in the case provided deep reflective learning, which led to changes to personal and team-wide practice. It has become the standard procedure to review all lymph node glass slides from specimens of colorectal resection staged as pT2 without lymph node metastases (pN0) or lymphovascular invasion detected on WSI. This measure prevents “understaging” and allows consideration of adjuvant therapy, should metastases or lymphovascular invasion be demonstrated. Future studies from other centres undergoing DP validation sharing their experiences will further help to elucidate potential challenges in DP and strategies to overcome them.

Of note, it may be that the discordance rate (1.3%) described by our group is an underestimate of the true discordance between digital and glass assessments. This limitation is inherent in the RCPath recommended method of validation as the pathologist self-verifies on glass and digital at the time of diagnosis and without blinding and/or dissociation between the two modalities it is not possible to capture the underlying true discordance rate. However, the RCPath validation process is intended to be pragmatic and able to be applied in real-world prospective validation settings. Previous studies have already published on the discordance rates using more rigid retrospective methodologies including wash-out periods between reviews and a meta-analysis, and systematic review of 25 studies found a 1.7% discordance rate [2]. A process that requires double reporting and/or re-review after a wash-out period would likely make the validation unmanageable for departments with time requirements for validation already highlighted as an issue (one comment).

### 4.4. Present and Future Perspectives

Validated consultants are now reporting on DP and refer to glass slides only when required on a case-by-case basis. We have an ongoing audit of 10% of digitally reported cases and continue to share our experiences of DP within our speciality teams. The laboratory still sends out glass slides in parallel with the digital images, and this will persist until every pathologist is fully validated. The benefits of a fully digitised workflow are yet to be seen, although positive impacts on quality and flexibility are already noted. This study does not aim to address matters of economy, workflow, or efficiency of DP compared to traditional glass-slide based diagnoses, which has been published elsewhere [3,24,25]. NHS Screening programme cases are currently not reported fully digitally, but national studies are underway to address the evidence gap in these programmes [26,27].

Pathologists’ confidence in reporting digitally compared with glass varied, but there were no trends by speciality. There are likely to be many factors influencing this, such as individual pathology experience and previous digital experience. Confidence is likely to also be influenced by personal approach to risk, and many pathologists reverted to glass for unusual or challenging cases. This is likely related to confidence in DP when faced with diagnostic uncertainty [28].

In this paper we focus on DP validation and its potential to leverage quality, efficiency, and flexibility benefits, however, it also provides the platform and infrastructure for deployment of AI for diagnostic assistance of pathologists. Validation for DP does not necessarily confer validation for use of AI and the process for this needs careful thought. Laboratories undertaking DP should aim for accreditation for this activity, for example in the UK this would be UKAS accreditation (ISO:15189) with an extension to scope for DP.

## 5. Conclusions

We have described our pan-departmental experiences of undergoing validation in DP covering a wide range of specialities and have demonstrated the real-world experiences and challenges of primary digital reporting. Whilst we have come across many of the established findings in DP and mitigated for them, we provide evidence of new insights across a greater breadth of specialities.

## Figures and Tables

**Table 1:** Duration on Stage 2 Validation.

Duration	Number of Pathologists
<6 months	5
6 months–1 year	3
12–18 months	8
> 18 months	4

**Table 2:** Live reporting validation statistics.

	Across All Specialities	Urology *	Breast	Renal	GI *	H&N	Skin	Gynaecology	Respiratory
Total no of cases	3777	566	660	138	1287	87	726	180	133
Technical deferral rate to glass	2.6%	2.1%	3.5%	0%	4%	5.8%	1.2%	0.6%	0%
Cases with discordances	1.3%	3.9%	1.5%	1.5%	0.8%	0%	0.8%	0%	0%

* Urology includes UGC, BKP and prostate. GI includes GI and Liver.

**Table 3:** Percentage out of total and number of cases with each type of discordance.

Type of Discordance	B1	B2	B3	N/A
Percentage of cases	0% (0/3777)	0.1% (3/3777)	0.4% (16/3777)	0.8% (30/3777)

Key: B1—serious error with fundamental aspects of the case. B2—significant error in supplementary parameter. B3—minor error in supplementary parameter. N/A—no clinical impact.

**Table 4:** Cases with discordances. Discordance key: B1—serious error with fundamental aspects of the case. B2—significant error in supplementary parameter. B3—minor error in supplementary parameter. N/A—no clinical impact.

Speciality	Pitfall	Free Text Comments, Including the Discordance Category
Urology Germ cell	Pagetoid spread into rete (one case)	B3. Case where pagetoid spread into rete missed on digital and seen on glass. Not clear if it was a digital issue as it was very subtle. Will know to look specifically for this in the future.
Prostate	Dysplasia identification (three cases)	N/A. Case where there was a possible focus of PIN on digital which was less convincing on glass. It was difficult on either platform so would require a glass check. N/A. Case of PIN seen digitally and need glass for confirmation. N/A. Case where digitally reported as benign and glass reported by another pathology as small foci of PIN and focus of ASAP.
	Subjective finding/Reported by another pathologist (four cases)	B3. Case where a tiny focus of tumour at the prostate base margin missed on digital but spotted on glass. Not clear if it was a digital issue as base is often harder to see tumour. No clinical difference. B3. Case with a minor difference in Gleason scoring—subjective. B3. Case of ASAP identified digitally and when reported by another pathologist identified as benign. N/A. Case where it was digitally reported as benign but on glass reported as ASAP by another pathologist
	No details included (one case)	N/A.
BKP	Muscularis propria identification (one case)	N/A. Case where pathologist missed presence of muscularis propria as first case reporting on digital and concentrating on tumour grade assessment. Diagnosis of tumour, grade and stage were correct and no impact on clinical management.
	Reactive atypia identification (two cases)	B2. Case deferred to check if it was benign due to the inflammatory changes.N/A. Case deferred to check florid reactive changes were definitely reactive as atypia stands out more on digital.
	Grading of dysplasia (two cases)	N/A. Case deferred to clarify G2 vs. G3. N/A. Case deferred to clarify high or low grade of small foci of papillary urothelial carcinoma amongst abundant radiotherapy changes.
	Grading (two cases)	B3. Case of minor difference in grading G2 versus G3. B2. Case of urothelial carcinoma falling short of high grade change digitally and reported as just meeting the criteria for high grade.
	Assessment of invasion (two cases)	B3. Case showed minor difference in staging from very suspicious TI versus T1. B3. Case reported digitally as Ta but reported on glass as early suspicious invasion by another pathologist
	Subjective finding (two cases)	B3. Case of PUNLMP versus low grade urothelial cell carcinoma, subjective difference. B3. Case of minor difference in grading between digital and glass. Subjective and reported by different pathologist.
	Unusual or complex case (two cases)	N/A. Case of complex bilateral renal tumours requiring glass for interpretation. N/A. Case was difficult required glass and opinions from other pathologists.
Breast	Mitotic count (three cases)	N/A. Case deferred as difficult to see mitoses on digital. Easy to find on glass and clearly enough to make the tumour grade 3. N/A. Case deferred as unable to score mitoses on digital. N/A. Case where slightly undercalled mitotic count on digitally. This did not affect the grade.
	Identification of dysplasia (one case)	N/A. Case of possible atypia in one duct Review of glass showed no atypia. Team opinion—agreed.
	Pleomorphism (one case)	N/A. Pleomorphism 3 on digital versus 2 on glass. This did not affect the grade.
	Calcium oxalate (one case)	N/A. Case deferred as could not see calcium oxalate confidently on digital and had to defer to glass to be able to polarise. Calcium phosphate is easy to see on digital.
	Her2 positivity amplified (three cases)	N/A. Case deferred as digital amplifies immunohistochemistry positivity which needs to be taken into account when assessing. B3. Case deferred as digital interpretation more difficult due to apparent enhanced intensity of immunohistochemistry. B3. Case deferred as assessed as just reaching 2+ on digital (with low confidence) but on glass staining clearly less intense and in category.
	No details included (one case)	N/A.
Renal	Necrosis identification (one case)	B3. Case where small area of eosinophilic necrosis was overlooked on digital slide, easier to spot on glass. I now know I need to raise the threshold of suspicion or adjust the image so that eosinophilic areas of necrosis are more readily spotted.
	Unusual or complex case (one case)	N/A. Case where diagnosis was deferred until after glass review due to limited experience with lesions of acute glomerular thrombotic microangiopathy on digital. Interestingly, mesangiolysis appeared crispier and microthrombi were more well defined on digital.
GI	Grading of tumour (one case)	N/A. Case with a slight difference in grade. Focal intermediate was better seen on glass, but also seen more clearly on unscanned slides.
	Helicobacter pylori identification (two cases)	N/A. Case where helicobacter pylori organisms were more easily visible on glass. Will need to always check glass if morphology suggests Helicobacter pylori but organisms not seen on digital.N/A. Case of very scanty H. Pylori organisms visible on toluidine blue.
	Identification of tiny focus of neuroendocrine tumour (one case)	N/A. Case deferred to check if a tiny focus of neuroendocrine tumour was present.
	Identification of metastatic focus (one case)	B2. Missed focus of metastatic disease in lymph node. This did not change the staging as other lymph nodes positive.
	Assessment of invasion (one case)	N/A. Case deferred as possible nodal involvement and adventitial involvement very focal and not definitely invasive tumour. Post-neoadjuvant tumour difficult on digital platform—need to see a few more of these before will be confident without review on glass.
	Mucin (one case)	B3. More mucinous differentiation identified on glass review. This changed the diagnosis to an adenocarcinoma with 40% differentiation to a mucinous adenocarcinoma. No difference to treatment or prognosis.
	No details included (two cases)	N/A.
Liver	Reactive atypia and assessment of invasion (one case)	N/A. Case where atypia more likely reactive on basis of appearance on glass slides (but difficult on both). Need glass review due to uncertainty with regards to invasion.
Skin	Mitotic count (one case)	N/A. Case deferred to count mitoses.
	Measurements of margins and Breslow thickness (three cases)	B3. Case with a slight difference in clearance margin but was not clinically significant. B3. Case with a closer margin as needed to see higher power on digital. B3. Case where Breslow thickness and deep margin changed on glass review.
	Incidental finding (one case)	N/A. Case where an incidental benign naevus was seen on glass which was not seen on digital. The main pathology was the basal cell carcinoma excision however and the parameters of the tumour were not affected so the clinical outcome did not change.

**Table 5:** Diagnostic features and skills that improved with experience.

Speciality	Learning Curve
General	Speed of reporting Navigating the slide Use of scanning magnification/panning for screening Realising when to slow down and what parameters to check Identifying potential pitfalls and when to defer to glass slides Grading of dysplasia Annotating lymph nodes
Urology Germ Cell	Assessment of rete invasion Assessment of hilar invasion Assessment of lymphovascular invasion Identification of GCNIS Identification of small foci of seminoma
Breast	Her2 scoring 1+/2+ cases
Renal	Identification of tubulitis in rejection and tubulointerstitial nephritis
GI	Identification of *Helicobacter pylori* on Toluidine blue staining
Respiratory	Identification of giant cells Counting IgG4-positive plasma cells

## Data Availability

All data available is presented in this paper. The validation summaries are confidential and cannot be made publicly available.

## Supplementary Materials

The following supporting information can be downloaded at: https://www.mdpi.com/article/10.3390/diagnostics13132144/s1, Figure S1: Cases with discordances (% of total cases); Table S1: Specialities of the histopathologists pathologists in this study; Table S2: Technical issues encountered; Table S3: Areas of potential pitfalls with the digital platform by speciality; Table S4: Diagnostic areas noted to be easier on the digital platform; Table S5. Key examples of situations where digital pathology assisted histopathologists; Table S6: Issues reported to have arisen due to use of the digital interface; Table S7: Pathologist’s diagnostic confidence; Table S8: ‘Results of Stage 2 pathologist views’. Diagnostic preferences of pathologists by speciality. Pathologist confidence scores on digital reporting vs. glass reporting; Table S9: Summary of all the data.
